# FR-CAPTCHA: CAPTCHA Based on Recognizing Human Faces

**DOI:** 10.1371/journal.pone.0091708

**Published:** 2014-04-15

**Authors:** Gaurav Goswami, Brian M. Powell, Mayank Vatsa, Richa Singh, Afzel Noore

**Affiliations:** 1 Indraprastha Institute of Information Technology, Delhi, India; 2 West Virginia University, Morgantown, West Virginia, United States of America; University of Leuven, Belgium

## Abstract

A Completely Automated Public Turing test to tell Computers and Humans Apart (CAPTCHA) is designed to distinguish humans from machines. Most of the existing tests require reading distorted text embedded in a background image. However, many existing CAPTCHAs are either too difficult for humans due to excessive distortions or are trivial for automated algorithms to solve. These CAPTCHAs also suffer from inherent language as well as alphabet dependencies and are not equally convenient for people of different demographics. Therefore, there is a need to devise other Turing tests which can mitigate these challenges. One such test is matching two faces to establish if they belong to the same individual or not. Utilizing face recognition as the Turing test, we propose FR-CAPTCHA based on finding matching pairs of human faces in an image. We observe that, compared to existing implementations, FR-CAPTCHA achieves a human accuracy of 94% and is robust against automated attacks.

## Introduction

A CAPTCHA is a Turing test designed to distinguish between humans and automated scripts [1]. These tests ensure that the user is a genuine person and not an automated script. CAPTCHAs can serve a variety of applications including but not limited to preventing: spam comments on blogs, automated fake registrations on website registration forms, automated voting in online polls, dictionary attacks on password systems, automated posting on forums and message boards, and automated usage of an online service beyond a specific threshold. Further, as detailed in Starvou et al. [2], under certain conditions CAPTCHAs can also be used to mitigate Denial of Service (DoS) attacks in combination with other security mechanisms. Since a large number of organizations and people rely on online services, DoS attacks and misuse of these services can have high negative impact and cause loss to the service provider. Therefore, measures against such attacks are important to ensure secure and reliable services. As a result, CAPTCHAs are widely used to protect online services such as email, web transactions, and mobile banking.

Current CAPTCHAs include several tests such as recognizing handwritten characters, differentiating between images of cats and dogs, and recognizing numbers or characters in a given audio/video segment. However, these CAPTCHAs suffer from various drawbacks. Text-based CAPTCHAs such as reCAPTCHA [3] require a user to decipher distorted text. Sometimes excessive distortion is used in these CAPTCHAs to secure against automatic attacks. Large amounts of distortions make these CAPTCHAs difficult and time-consuming for a genuine user to solve. Although there is provision to request a new instance of the CAPTCHA, this interrupts the user experience.

In addition, text-based CAPTCHAs inherently suffer from alphabet and language dependencies. Most of these are designed using the English alphabet which may be easily solved by a native English speaker. However, users belonging to different demographics (non-English native language) can face difficulty in solving these CAPTCHAs. Von Ahn et al. [3] and Bursztein et al. [4] showed concrete evidence of such dependencies of the CAPTCHA. Since web services are not limited to regions with English-speaking populations, addressing these limitations is important for designing a widely usable CAPTCHA. Image-based CAPTCHAs such as IMAGINATION [5] and Asirra [6] do not demonstrate any such dependence. However, both of these have been solved successfully by automatic algorithms and hence are vulnerable to attacks [7], [8]. IMAGINATION does include an annotation step to provide an additional security layer against bots. However, this step involves identifying the category of a displayed image from several presented options and the user is required to possess a decent English vocabulary in order to solve it. Therefore, IMAGINATION also suffers from language and alphabet dependencies similar to reCAPTCHA.

Video [9] and audio-based [10] CAPTCHAs have also been explored in literature. However, video CAPTCHAs require higher bandwidth compared to text and image CAPTCHAs. Traditionally, the size of a text/image-based CAPTCHA is less than a few kilobytes whereas video CAPTCHAs might easily be several megabytes in size. Therefore, they are not ideal for situations where bandwidth is scarce, such as in developing countries and also in cases when the user is utilizing 2G networks or usage-limited costly 3G networks. In such scenarios, incurring additional data usage charges for a security measure is not acceptable to users. While audio CAPTCHAs provide a means to account for visually impaired users, they require sound output to be enabled on the device which may not always be possible. Audio CAPTCHAs add background noise to the encoded message which makes it difficult to decipher without good sound quality and sufficient clarity. Also, some users may face difficulty in understanding the message regardless of noise because of unfamiliarity with the accent in which the audio message is communicated.

This research explores the possibility of using face recognition to address the challenges with existing CAPTCHAs. Face recognition is a highly intuitive task for humans that can be used as a Turing test. Unlike the aforementioned tests, it does not suffer from language dependency. The human mind performs these functions every day and is very effective in recognizing human faces. However, even after decades of research in face detection and recognition, there exist several challenges in designing effective and accurate algorithms for automatic face detection and recognition. The distortions such as rotation, noise, blur, blending with the background, and occluding facial features can cause face detection algorithms to falsely reject a face. Automatic face detection algorithms are also unable to accurately distinguish between synthetically generated faces and human faces. The results of the Multiple Biometric Evaluation (MBE) [11] show that state-of-the-art face recognition algorithms yield good performance for controlled face recognition. However, their performance is greatly reduced when the images are captured in an uncontrolled environment with variations such as pose and inconsistent image quality [12]. In contrast, research in cognitive science has shown that humans are good at recognizing familiar faces even if they are of poor quality and distorted [13]. Recent research has shown that under partially controlled conditions and with sufficient image size, even in the case of unfamiliar faces, humans perform better than automatic algorithms [14]. Our previous work explores the possibility of using only face detection to design a CAPTCHA [15]. In this research, our hypothesis is: *“*A CAPTCHA that requires face detection and recognition should be challenging for automatic algorithms while being simple for humans to solve.” Since the research in design of CAPTCHAs has focused on developing tests that are easy for humans but difficult for machines, incorporating challenging face detection and recognition tests in CAPTCHA can enhance their security.

## Materials and Methods

### Ethics Statement

The human study is conducted with the help of volunteers with 18+ years of age. Prior to collecting human responses, the written consent of the volunteers is obtained and they are informed that their responses would be used for research and analysis purposes. Names or any other identifiable information of the volunteers are not collected. All the procedures used in the current study are approved by the IIIT-Delhi Ethics Board.

### Stimuli

The face images used in the CAPTCHAs are taken from the AR face database [16] which allows use for research and publication with proper citation. The non-face images are available under the Creative Commons license and are taken from various sources on the internet.

### Methodology

**Figure 1:**
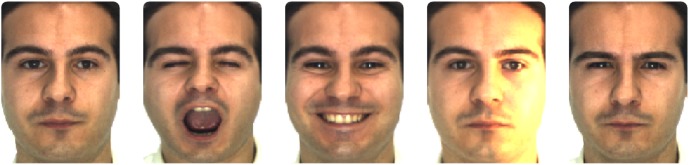
Natural variations present in face images. Variations such as expression and illumination are common even when pose of the face image is fixed to be frontal. The performance of automated face recognition attack algorithms suffers in the presence of such natural variations whereas these are easy to process for humans.

Face recognition is a problem that every healthy human mind solves everyday, usually even without conscious effort. Cognitive research has found that human face recognition can function even in the presence of a multitude of covariates such as pose, expression, illumination, occlusion, aging, alterations and their combinations [13]. On the other hand, automated face recognition algorithms perform poorly when such challenges are present. The proposed CAPTCHA leverages this parity by utilizing distortions and natural variations of human faces to increase difficulty for automated attacks. Natural face variations such as shown in Figure 1 are easy for the human mind to process but difficult for automated algorithms. In addition to natural variations, the task can be made even more challenging by applying artificial distortions to the face images and embedding these on a complex background. However, adding artificial distortions may adversely impact both machine and human performance. As discussed previously, an ideal CAPTCHA should be easy for humans to solve while being robust towards automated algorithms. Therefore, the artificial distortions are applied within certain limits based on the sets of parameters. These distortion parameter sets have to be optimized in order to achieve the best tradeoff between ease for human users solving the CAPTCHA and difficulty for automated algorithms.

The proposed CAPTCHA is a single composite image containing multiple human face and non-face images on a background with varying degrees of distortion. In order to solve it, the user is required to match human faces (faces belonging to a single individual) on the CAPTCHA. The proposed FR-CAPTCHAs are designed using an adversarial learning-based algorithm. Since the objective of the CAPTCHA generation process is to maximize human accuracy while minimizing machine accuracy, automatic face recognition algorithms act as the adversary in the proposed approach. The CAPTCHA generation process depends on several parameters that need to be optimized. The FR-CAPTCHA generation process can be formulated as,
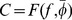
(1)Here, 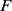 is a function that utilizes face and non-face images, 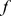, along with the sets of parameters 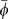, to generate the FR-CAPTCHA denoted by 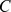. The sets of parameters control the difficulty of the generated CAPTCHA. Using an adversarial learning approach and gradient descent optimization, the optimum sets of parameters are obtained such that the human accuracy is maximized and machine accuracy is minimized. In order to achieve this objective, the CAPTCHA generation process is divided into two different processes: learning the optimum sets of parameters, and utilizing the learned sets of parameters to generate new FR-CAPTCHAs. The useful sets of parameters 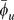 are learned according to Equation (2):
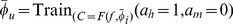
(2)where, 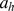 denotes the human response and 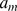 denotes the machine response. A correct response is depicted with the value 1, whereas an incorrect response is depicted with the value 0. Out of all the possible parameter sets 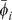, only the useful parameter sets 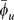 are chosen according to the specified constraint on 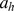 and 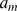. It is to be noted that the constraint denotes the ideal criteria that a CAPTCHA has to fulfill: maximum human performance and minimum machine performance. Therefore, parameter sets conforming to other constraints are not useful for FR-CAPTCHA generation. Further, in Equation (2), the process Train represents gradient descent learning. For a given set of parameters 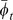, let 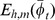 be the objective function that minimizes the error caused by four constraints associated with 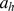 and 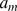 i.e.,



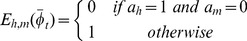
(3)In gradient descent, optimal parameters are obtained using Equations (4) and (5).
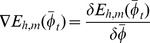
(4)

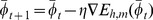
(5)Here, 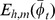 denotes the gradient of the objective function at the 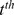 learning iteration and 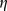 is the learning rate used to control the rate at which parameter learning converges. The initial parameter assignment determines the final outcome of gradient descent learning. Therefore, the initial assignment is performed using small scale experiments with both humans and automatic algorithms. An intermediate experiment is performed in order to obtain human responses. The details of the experiment are presented in the results section. Since the CAPTCHA performance depends on both human and machine responses, machine adversaries are also required to be analyzed. As FR-CAPTCHA is based on face detection and recognition, automatic face detection and recognition algorithms are suitable adversaries. Therefore, machine adversaries based on the Viola-Jones face detector [17] and a commercial face recognition system (COTS) are used. The methodology involved with the adversaries is detailed below:

1. Given a CAPTCHA image 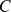, a set of images 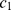 to 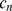 are created by rotating the CAPTCHA image by 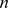 incremental rotation levels ranging from 0° to 360° degrees in order to handle the random rotation in the constituent images.
2. For each CAPTCHA image 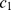 to 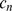, the Viola-Jones face detector [17] is utilized and face detection is performed with three different window sizes (window size is a parameter of Viola-Jones face detector).
3. The detected face coordinates are matched with the actual embedded face locations. The number of correctly located face images is stored and the number of all unique faces detected from the CAPTCHA is obtained by summation over the set of images 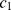 to 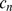. For example, if a face image was only correctly identified in 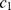 but not in any other images, it is still counted as a successful detection.
4. If the cumulative successful face image detections equal the actual number of human faces in the CAPTCHA, it is considered as a correct response by the face detection adversary, otherwise it is not.
5. The methodology of the machine adversary based on face recognition COTS is the same as the one based on Viola-Jones detector. The difference between the two is that in Step 2, face detection is performed using the COTS face detection module instead of Viola-Jones. If COTS is able to detect two face images, then recognition module is invoked to match them. Face recognition adversary is successful if it is able to detect at-least two faces and find a correct face pair.

As per Equation (5), the learning process considers both the human response (*h*) as well as the response of the automatic algorithm (*m*). The gradient descent learning converges to multiple optimal sets of parameters that maximize the likelihood of being solved successfully by humans and minimize the likelihood of being solved successfully by automatic algorithms. The optimal sets of parameters are then utilized in the testing phase of FR-CAPTCHA.

**Figure 2:**
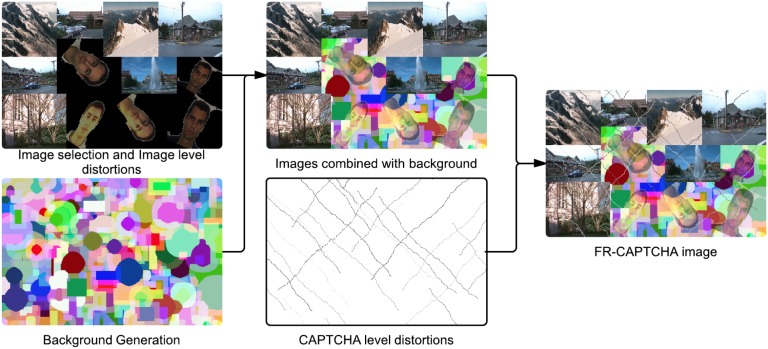
The FR-CAPTCHA generation process. An image of the same size is created using a randomly selected set of human faces and other non-face images after applying different amounts of rotation to each image. A background of the same size is generated using various colored shapes. A new image is generated by blending this image with the background. This combined image is then further processed to add noise, illumination variance, and false edges. The resulting image is a FR-CAPTCHA.

Figure 2 shows the steps involved in generating the proposed FR-CAPTCHA based on the optimum sets of parameters. The optimum parameter sets describe the range of parameter values for the various operations that are essential to the generation process. For example, a parameter set can specify that rotation angle has to lie within the interval 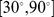. This means that even though the final rotation applied to each image in the FR-CAPTCHA is chosen in a stochastic manner, it is bound to lie between the specified upper and lower limits. First, a set of human face and non-face images is selected from a database of images. The number of human face and non-face images can vary in individual instances of FR-CAPTCHA and is randomly decided at the time of generation. The only constraint in selecting face images for the CAPTCHA is that there must be at least two matching face pairs. A matching pair of faces is one in which both faces belong to the same individual, but are not the exact same image. Each selected image is resized according to the parameter set and then positioned on a blank image of size 600
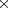
400. Since modern devices with varying screen sizes are used to access the internet, the choice of size is an important factor. This size preserves the details required by a human to successfully perform face recognition without being too large for devices with small screens, e.g., mobile and tablet devices. The images are placed at randomly selected positions such that they do not overlap beyond a specified threshold and the overlapping region is created using a weighted average of the overlapping images. We select multiple distortions for this purpose such as rotation, noise, illumination, false edges, adding non-face images, and image blending with the complex background. Further, to maintain unpredictability, each of these artificial distortions is applied in a stochastic manner but with an intensity that is regularized by the optimum parameter sets. The parameters control the difficulty of the generated CAPTCHA. After placing all the component images, a background image of the same size (600
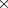
400) is generated. To generate the background image a large number of shapes, varying in size, color and type (circle, rectangle, cross, ellipse), are placed on an image. Patches of skin color are also placed on the background to obfuscate skin color-based segmentation algorithms. Using a weighted average blending scheme, the background is combined with the image containing rotated and resized faces obtained in the first step.

**Figure 3:**
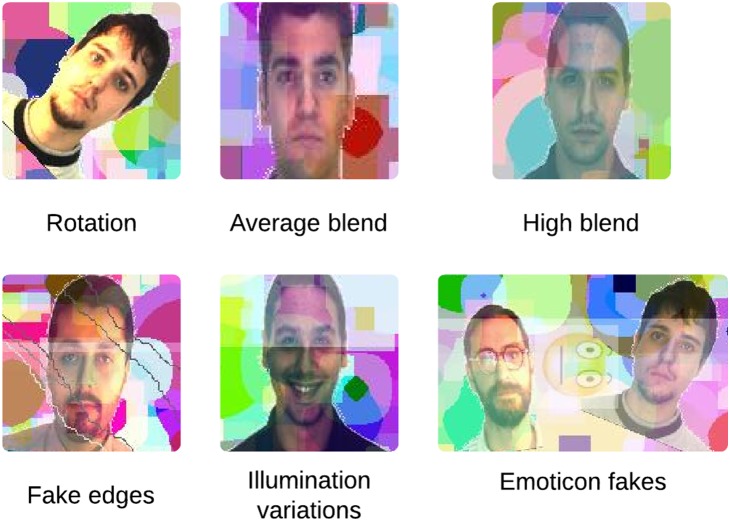
Distortions involved in the FR-CAPTCHA generation process.

To further enhance the security of the CAPTCHA, irregular variations in illumination are introduced to the image. In addition, a random amount of free-form lines are drawn on the image to introduce false edges and emoticon images are blended in with the CAPTCHA at dynamically selected locations. For any machine adversary, the first step towards solving the CAPTCHA is segmentation into distinct sections so that the embedded faces can be extracted for matching. Adding false edges and emoticons introduces false positives for edge and face detection algorithms respectively, and therefore, increases the security of the generated CAPTCHA. The distortions utilized in generating FR-CAPTCHA are illustrated in Figure 3. The complete step-by-step process of generating a FR-CAPTCHA is summarized below:

1. Image selection and image level distortions
  - The number of human faces to place on the CAPTCHA is decided. This number can vary from 4 to 6.
  - The number of non-human images in the CAPTCHA is set such that the total number of faces in the CAPTCHA is 12.
  - The required number of face and non-face images are randomly selected from the corresponding databases.
  - Each selected image is resized to a size that can range from 100
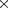
125 to 175
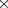
150.
  - Each selected image is rotated by an angle that is stochastically chosen such that it lies within a range specified by the parameter set.
  - For each image, a random coordinate (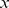, 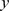) is generated. Then a check is performed to verify if the image can fit inside the CAPTCHA boundaries if (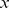, 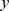) is used as the top-left corner of the image.
  - Check if the image overlaps with an existing image on the CAPTCHA. If so, both images are blended together using weighted average blending. The weights depend on whether or not the overlapping image is a face. The face image is assigned higher weight in the blending process compared to the non-face image in order to facilitate human recognition. These weights are a part of the parameter set of FR-CAPTCHA generation which is subject to optimization.
  - Repeat steps (d), (e), (f) and (g) until all 12 selected images have been placed.
2. Background generation
  - An image of size 600
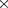
400 is generated.
  - A number of shapes such as squares, crosses, and ellipses are generated with varying sizes and colors. These variations, as well as the amount of shapes to be generated, are controlled by respective parameters which specify the range of values that these parameters may take.
  - These generated shapes are placed randomly on the background image without consideration for overlap.
  - Finally, the background image is eroded and dilated with varying structuring elements to generate the final background for the FR-CAPTCHA.
3. Combining background with CAPTCHA image
  - The background image is blended with the CAPTCHA image using weighted averaging blending. The weights assigned to the background and foreground (CAPTCHA image) are part of the parameter set.
  - The weight assigned to the foreground is higher in the regions where there already exists an overlap of face images prior to combination with the background. This is to ensure that every region in the FR-CAPTCHA is discernible by humans.
4. The resultant CAPTCHA image (foreground with selected human and non-human images and background) is subjected to further distortions based on the parameter set. These distortions include:
  - Adding fake edges: Irregularly oriented jagged lines with flexible starting and ending locations.
  - Adding illumination variations: The CAPTCHA image is divided into a variable number of non-uniform grids. These grids are then subjected to gamma adjustment with different gamma values such that some grids are made lighter and some are made darker.
  - Adding emoticon images: A database of emoticon images is used to select and place few emoticon images on random locations in the CAPTCHA image. These emoticon images serve as false positives for face detection algorithms and make segmentation of the individual faces difficult.
  - Adding noise: A certain percentage of the total number of pixels in the FR-CAPTCHA are corrupted by modifying their values. The percentage of pixels to corrupt is decided on the basis of the parameter set and their locations to corrupt are chosen at random.

**Figure 4:**
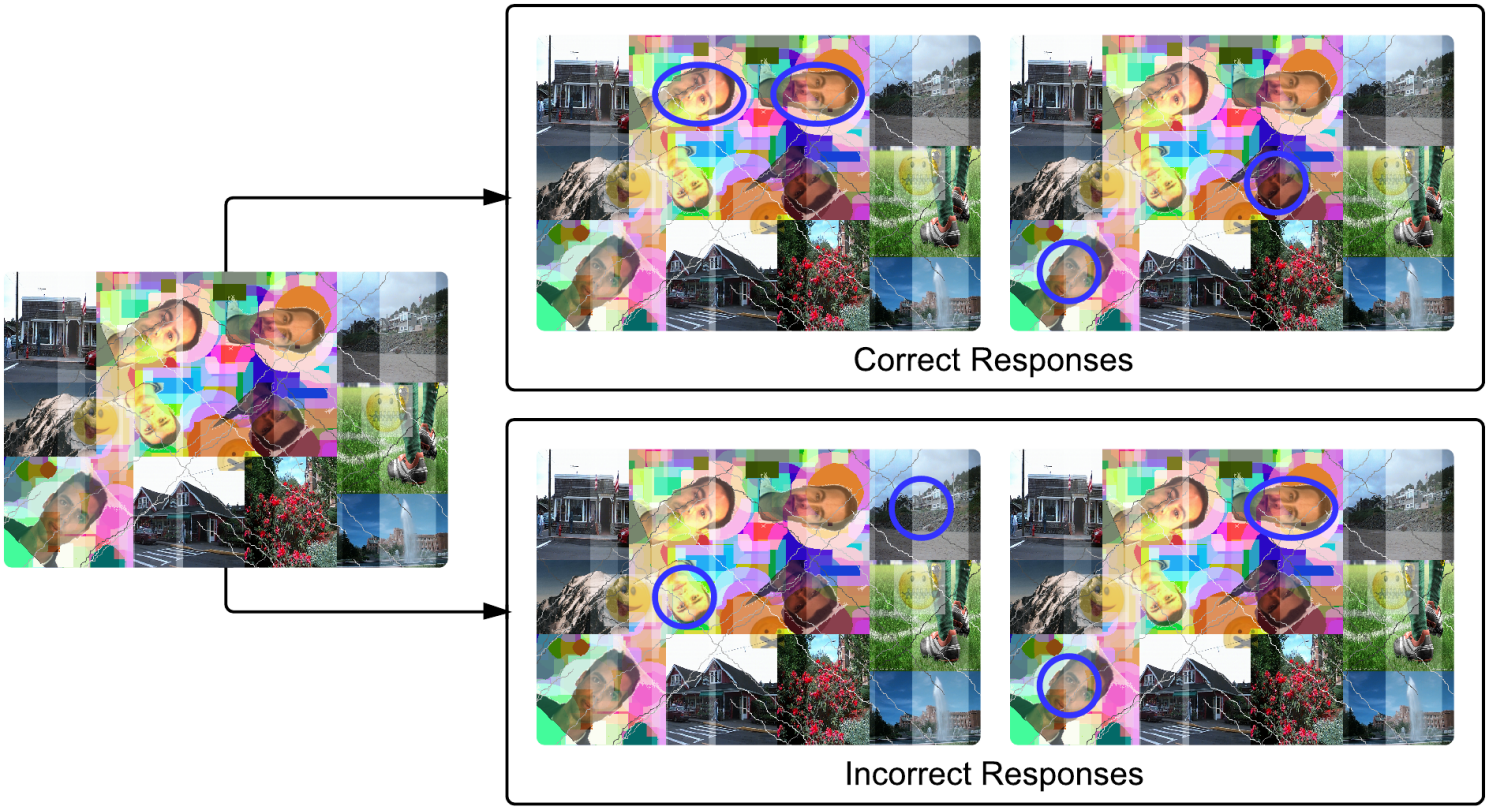
Example of correct and incorrect responses to the proposed FR-CAPTCHA. It can be solved correctly if and only if a matching pair of human faces is selected by the user. Each blue circle represents a user response. As shown, selecting two face images of the same person is considered as a correct response, while selecting a non-face image or two images of different persons is considered an incorrect response.

Figure 4 presents an example of FR-CAPTCHA along with sample correct and incorrect responses. As mentioned previously, every FR-CAPTCHA contains at least two pairs of faces belonging to the same individual. In order to solve the test and verify that the user is human, he/she must mark the locations of two human faces in the image which belong to any one of the matching pairs. If any of the marked locations is not a human face or the faces marked belong to different individuals, the response is considered incorrect; otherwise, it is considered correct and the CAPTCHA is considered solved. The process of solving the FR-CAPTCHA involves both detecting the locations of human faces in the image as well as recognizing which faces match with others in the image. The FR-CAPTCHA thus requires a user to solve both face detection and recognition problems. The process of solving a FR-CAPTCHA is also detailed below:

1. Each FR-CAPTCHA has a total of 12 images. Only 6 (at most) of these images can be human faces. Every user is allowed 2 clicks in total to solve a FR-CAPTCHA. For an attempt to be called successful, the following conditions must hold true for the 2 clicks made by the user:
  - Both the clicks have to be located within the area of human face images. If any of the clicks lie in a region which is part of the background or a non-human face image, then the attempt is invalid. This is the face detection test. Among the incorrect responses shown in Figure 4, the first response violates this condition.
  - Both the human face images selected by the user (by clicking in their region) have to belong to the same individual. If both images are human faces but belong to different individuals, then the attempt is invalid. Each FR-CAPTCHA has at least two correct ways to be solved, i.e., there are 2 pairs of face images that belong to the same individual. The user has to pick any of these pairs. This is the face recognition test. Among the incorrect responses shown in Figure 4, the second response violates this condition.

## Results and Discussion

FR-CAPTCHA is evaluated in two phases. The first phase of testing is performed during parameter optimization in order to obtain human and machine adversary responses for gradient descent learning. The second phase of testing is performed on the optimized FR-CAPTCHA in order to assess its robustness towards automated attack as well as ensure ease of use for humans. The following sections provide the details of these results.

### Parameter Optimization

As discussed in the previous section, human responses are required in order to optimize the set of parameters for FR-CAPTCHA generation. An initial set of FR-CAPTCHAs is generated with multiple sets of parameters and human performance is evaluated on these sets. The parameter values are explained in Table 1 and 10 parameter sets on which the optimization/evaluations are performed are listed in the Table 2. A total of 300 CAPTCHAs are generated - 30 CAPTCHAs for each parameter set. Human performance on each set are presented in Table 2. During the human performance test, each CAPTCHA is presented to multiple volunteers. Each CAPTCHA is successfully solved by at least one attempting volunteer. Human performance data on these sets provides valuable insight into the impact of each distortion towards human accuracy. For example, the observation that human performance is lower in set 10 compared to set 9 implies that adding emoticon images and high global distortions negatively impact human performance. Therefore, it becomes clear that increasing the level of these distortions corresponds to a proportionate increase in the difficulty of the entire CAPTCHA. This deduction can be supported by the results of other sets and relationships between the distortions and CAPTCHA difficulty can be established. Tuning these distortions optimally then becomes relatively easier. In order to achieve maximum human accuracy, the distortions must be chosen such that the CAPTCHA is easy to solve, however, it must still be resilient against machine attack and therefore a balance has to be achieved via the optimization process. During this phase, the key observations are as follows:

**Table 1:** Range of Parameter values.

Parameter	Value
No rotation	0°
Low rotation	0°–60°
Medium rotation	30°–120°
High rotation	45°–170°
No blend	100% foreground
Low blend	80% foreground
Medium blend	65% foreground
High blend	50% foreground
No global distortions	illumination = false, false edges = false
Low global distortions	either illumination or false edges
High global distortions	illumination = true, false edges = true

**Table 2:** Human performance for each set of CAPTCHAs during parameter optimization phase.

Set No.	Distortion	Human Accuracy (%)
1	No rotation, no blend, no global distortions, no emoticons	100
2	Low rotation, no blend, no global distortions, no emoticons	100
3	Low rotation, low blend, no global distortions, no emoticons	100
4	Low rotation, low blend, low global distortions, no emoticons	100
5	Low rotation, low blend, low global distortions, emoticons	100
6	High rotation, low blend, low global distortions, no emoticons	100
7	Low rotation, high blend, no global distortions, no emoticons	79.2
8	Medium rotation, medium blend, no global distortions, emoticons	90.0
9	Low rotation, low blend, low global distortions, no emoticons	100
10	Low rotation, low blend, high global distortions, emoticons	92.0

1. Overall, across all the sets, 96% training accuracy is achieved using 1794 responses from 220 volunteers.
2. Even though human performance is high across all the sets, reduced accuracies are observed when the blending level is high (79.2% in set 7). The blend effect causes the visibility of the face image to deteriorate significantly, especially due to the overlapping images and highly randomized background. Therefore, it is noted that the maximum level of blend requires to be reduced in the optimized set of parameters.
3. It is also observed that rotation, global distortions and emoticons have relatively smaller negative impact and these distortions can be utilized without compromising the human performance.
4. For easier sets, automatic face detection algorithms (adversaries) are able to detect some faces in the CAPTCHA images. However, with increase in distortions, automatic algorithms are unable to find genuine faces.

### Human Performance Evaluation of FR-CAPTCHA

**Figure 5:**
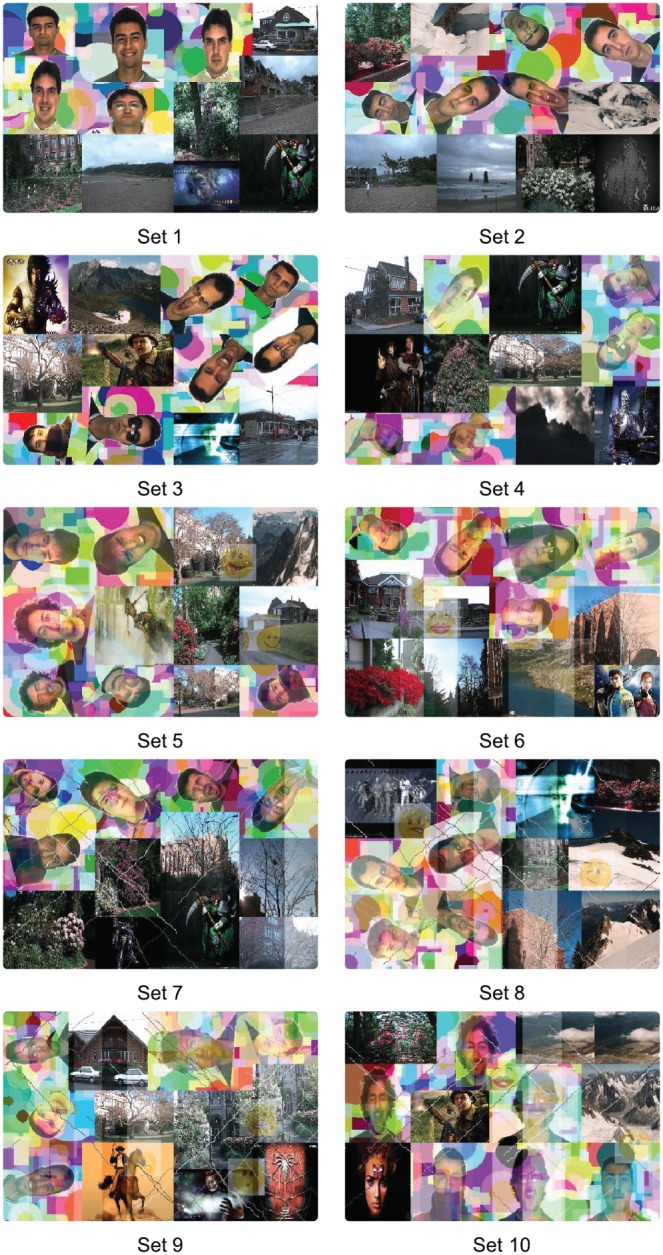
Examples of FR-CAPTCHA. The CAPTCHAs are numbered according to the set numbers assigned in Tables 1 and 2.

For evaluation, a set of 500 CAPTCHAs are used with varying difficulty and collected responses in two sessions in different demographic settings (i.e., India and the United States). Some key observations are as follows:

1. In session 1, we collected 30,000 responses from 3,000 users with an overall accuracy of 98.36% (29,507 correct responses). In session 2, we collected 12,000 responses from over 1,200 users with an overall accuracy of 84.89% (10,187 correct responses). In both sessions, each CAPTCHA image is shown to multiple volunteers. The CAPTCHAs are presented to the users on a webpage where they are provided general instructions on how to solve a FR-CAPTCHA.
2. Using a weighted average based on the fraction of responses contributed by each session (30,000 responses in session 1 and 12,000 responses in session 2), we get an overall human accuracy of 94% over the two sessions.
3. We also observe that performance depends on the difficulty level of the CAPTCHA. The CAPTCHAs used for evaluation corresponded to ten different difficulty levels. Table 3 provides detailed per-set analysis of human performance on these CAPTCHAs. Set 1 is the easiest set with no distortions, whereas, Set 10 is the hardest set with the maximum amount of distortion. As we can see in Table 3, for a particular session, human accuracy does not decrease by a large amount even with considerable increase in distortion across various sets. The difference between the best and worst performance is only 6.6% in session 1 and 7.1% in session 2. This supports our initial hypothesis that humans can recognize faces even in unfavorable conditions with unfamiliar faces. Though the difference in the overall performance in both sessions is much higher, a part of this can be attributed to the difference in the conditions during response collection. Another inference that can be drawn from the set analysis is that out of all the distortions, blending with background has maximum effect on human performance. This is evident from the performance values in Sets 3, 7, and 9 which are clearly much lower than the sets of comparable difficulty, with similar amount of distortions. Figure 5 presents an example FR-CAPTCHA image belonging to each of these sets.
4. Another interesting observation is that the face images utilized in the experiment belong to the AR Face database which mainly comprises Caucasian subjects. The Other Race Effect (ORE), which has been explored in face recognition literature, suggests that volunteers from the Caucasian race should perform significantly better than non-Caucasian volunteers. Our results show that, for solving FR-CAPTCHA, Indian volunteers have overall better performance and ORE is not observed. Since the FR-CAPTCHA test only involves differentiating between two pairs of face images, the impact of ORE is minimal in this scenario. Further, in order to minimize the possibility of human failure, the selection of the face dataset utilized to generate FR-CAPTCHA for a particular web resource can be easily adjusted according to the ethnicity of the primary target audience.

**Table 3:** Human performance for each set of CAPTCHAs.

Set No.	Distortion	Human Accuracy (%)	
		Session 1	Session 2
1	No rotation, no blend, no global distortions, no emoticons	100	88.6
2	Low rotation, no blend, no global distortions, no emoticons	100	86.5
3	Low rotation, high blend, no global distortions, no emoticons	100	83.9
4	Medium rotation, low blend, no global distortions, no emoticons	100	86.4
5	Medium rotation, no blend, low global distortions, no emoticons	100	85.3
6	Medium rotation, low blend, low global distortions, no emoticons	99.2	83.3
7	High rotation, medium blend, medium global distortions, no emoticons	95.4	81.8
8	High rotation, low blend, medium global distortions, emoticons	97.6	83.2
9	High rotation, high blend, high global distortions, emoticons	93.4	81.5
10	High rotation, low blend, high global distortions, emoticons	94.4	84.4

### Comparison with reCAPTCHA and IMAGINATION

**Figure 6:**
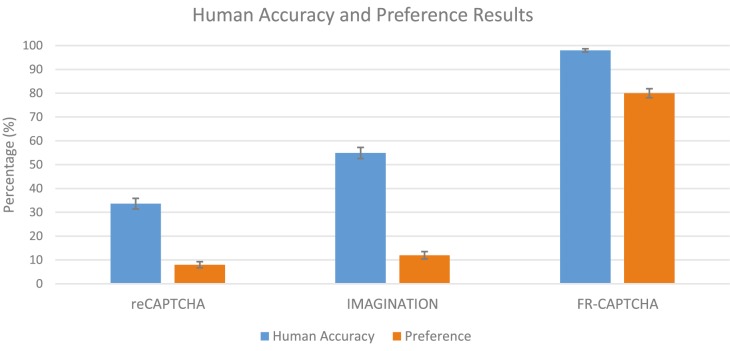
Human performance and preference on reCAPTCHA, IMAGINATION and the proposed FR- CAPTCHA. Based on 2,997 responses, the proposed CAPTCHA achieves higher human performance and is also preferred by majority (80%) of the users. The error bars represent confidence intervals.

In addition to FR-CAPTCHA, human performance is evaluated on two other CAPTCHAs, the popular reCAPTCHA and IMAGINATION. A majority of the volunteers were not native English speakers. For each of the three CAPTCHAs, 2,997 responses were collected. Out of these, 1,008 responses were correct for reCAPTCHA, 1,646 responses were correct for Stage 1 of IMAGINATION, and 2,937 responses were correct for the proposed FR-CAPTCHA. Each user attempted one each of these three CAPTCHAs and was asked his/her preference based on the ease with which they could solve these tests. 80% of the users preferred the proposed FR-CAPTCHA over reCAPTCHA and IMAGINATION which were preferred by 8% and 12% of the users respectively. The results of this evaluation are presented in Figure 6.

### Performance of FR-CAPTCHA against Automated Attack

Besides efficient human performance, a CAPTCHA must also be resilient towards automatic attacks. Since the first step towards breaking a CAPTCHA would be to detect and segment the faces from the entire image, face detection tests were executed on the FR-CAPTCHAs using the above mentioned machine adversaries, but all of the face images were not detected correctly in any of the test CAPTCHAs. In addition, a script designed to find bounding boxes of objects embedded in CAPTCHAs which is successfully able to decipher the objects in IMAGINATION CAPTCHA, was not able to locate all of the objects in any face recognition CAPTCHA [7]. Also, the probability of correctly solving a face recognition CAPTCHA by a random guess method is approximately 0.0069 or 7 in 1000. A detailed computation is presented below:

The number of pixels in a FR-CAPTCHA of size 600
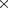
400 is 240,000. If we assume a constant size of each face image (100
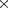
100), for a correct response the automated algorithm has to guess a pixel belonging to a face. Since there are two matching pairs of genuine faces (i.e. total of 4 face images pertaining to 2 individuals), the probability of this guess being correct is: 4
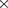
100
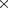
100/240,000 = 0.167. The next guess has to lie in the face region of the matching face failing which it will be an invalid guess and the entire attempt will fail. Therefore, since only a 100
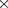
100 region is the favorable outcome, the probability of this guess being correct is: 100
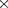
100/239,999 = 0.0417. Note that here, the previous pixel will not be counted for a valid guess and hence the number of possible outcomes (pixels) becomes 239,999. Also, since it may so happen that the pixel in the first case was chosen on the edge of the face region, the immediately adjacent pixel might contain the matching face. Therefore, even adjacent pixels remain possible candidates for selection in this step and the number of possible outcomes is not reduced any lower than 239,999. Following these calculations, the possibility of the entire attempt being correct is: 0.167
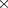
0.0417 = 0.0069 or approximately 7 in 1000.

It should be noted, however, that this calculation assumes that all face regions are of the same size, which is not the case. Therefore, the above calculation can only be considered as an approximation of the probability that a random guess can solve any given FR-CAPTCHA image.

## Conclusion

In this paper, a novel CAPTCHA is proposed which utilizes face recognition and detection as the Turing test. By analyzing the results obtained using two demographically diverse groups of volunteers, we can assert that the proposed CAPTCHA is user-friendly and easy to solve. Moreover, it is robust and secure against automated attack. It addresses the language/alphabet dependency challenges of text-based CAPTCHAs while remaining intuitive and simple for human users. In addition, the success of the proposed CAPTCHA also confirms that humans can match faces under severe distortion with high accuracy. We believe that the proposed face recognition CAPTCHA facilitates security against bots in online services without compromising user convenience.

**Additional Information:** Website for FR-CAPTCHA: http://research.iiitd.edu.in/groups/iab/frcaptcha.html.
